# 3D grating-based X-ray phase-contrast computed tomography for high-resolution quantitative assessment of cartilage: An experimental feasibility study with 3T MRI, 7T MRI and biomechanical correlation

**DOI:** 10.1371/journal.pone.0212106

**Published:** 2019-02-14

**Authors:** Julia Herzen, Dimitrios C. Karampinos, Peter Foehr, Lorenz Birnbacher, Manuel Viermetz, Rainer Burgkart, Thomas Baum, Fabian Lohoefer, Moritz Wildgruber, Franz Schilling, Marian Willner, Mathias Marschner, Peter B. Noël, Ernst J. Rummeny, Franz Pfeiffer, Pia M. Jungmann

**Affiliations:** 1 Chair of Biomedical Physics, Department of Physics and School of BioEngineering, Technical University of Munich, Garching, Germany; 2 Department of Radiology, Klinikum rechts der Isar, Technical University of Munich, Munich, Germany; 3 Department of Orthopaedics and Sportsorthopaedics, Klinikum rechts der Isar, Technical University of Munich, Munich, Germany; 4 Department of Neuroradiology, Klinikum rechts der Isar, Technical University of Munich, Munich, Germany; 5 Translational Research Imaging Center, Department of Clinical Radiology, Universitaetsklinikum Muenster, Muenster, Germany; 6 Department of Nuclear Medicine, Klinikum rechts der Isar, Technical University of Munich, Munich, Germany; 7 Institute for Advanced Study, Technical University of Munich, Garching, Germany; 8 Department of Radiology, University Hospital Freiburg, Freiburg, Germany; University of Umeå, SWEDEN

## Abstract

**Objective:**

Aim of this study was, to demonstrate the feasibility of high-resolution grating-based X-ray phase-contrast computed tomography (PCCT) for quantitative assessment of cartilage.

**Materials and methods:**

In an experimental setup, 12 osteochondral samples were harvested from n = 6 bovine knees (n = 2 each). From each knee, one cartilage sample was degraded using 2.5% Trypsin. In addition to PCCT and biomechanical cartilage stiffness measurements, 3T and 7T MRI was performed including MSME SE T2 and ME GE T2* mapping sequences for relaxationtime measurements. Paired t-tests and receiver operating characteristics (ROC) curves were used for statistical analyses.

**Results:**

PCCT provided high-resolution images for improved morphological cartilage evaluation as compared to 3T and 7T MRI. Quantitative analyses revealed significant differences between the superficial and the deep cartilage layer for T2 mapping as well as for PCCT (P<0.05). No significant difference was detected for PCCT between healthy and degraded samples (P>0.05). MRI and stiffness measurements showed significant differences between healthy and degraded osteochondral samples. Accuracy in the prediction of cartilage degradation was excellent for MRI and biomechanical analyses.

**Conclusion:**

In conclusion, high-resolution grating-based X-ray PCCT cartilage imaging is feasible. In addition to MRI and biomechanical analyses it provides complementary, water content independent, information for improved morphological and quantitative characterization of articular cartilage ultrastructure.

## Introduction

Osteoarthritis affects millions of individuals in the aging society, causing increasing socioeconomic challenges [1]. Articular cartilage lesions represent a major cause for early osteoarthritis [2, 3]. Therefore, early detection of cartilage degeneration is most important before irreversible cartilage loss occurs [4]. Optimal biochemical composition and integrity of articular cartilage matrix, mainly consisting of collagen fibers, proteoglycans, and water, is essential for optimal viscoelastic properties and function [5–7].

Several quantitative cartilage magnetic resonance (MR) imaging techniques have been applied in clinical research studies [5, 8]. T2 relaxation time measurements have been shown to correlate with collagen disruption and increasing intracartilaginous water contents and to predict cartilage loss [9, 10]. Correlations of T2 relaxation times with biomechanical properties were demonstrated at 1.5T and at 9.4T [11, 12], but not at 3T and 7T despite its clinical relevance.

Alternatively, CT or CTA may be performed in order to detect cartilage lesions in patients with cardiac pacemakers or claustrophobia or for improved detection of cartilage defects in thin cartilage layers [13]. However soft tissues provide rather low tissue contrast in conventional multi-slice CT [14]. Quantitative cartilage imaging using CT has only been successful using intraarticular contrast media [15]. In contrast to attenuation of X-rays in conventional CT, phase-contrast computed tomography (PCCT) uses the physical effect of refraction resulting in a phase shift, which x-rays experience while passing through matter, as a contrast mechanism. Although several phase-contrast methods exist, grating-based PCCT has been demonstrated to be efficiently compatible with standard, polychromatic X-ray sources [16, 17]. It provides a high-resolution three-dimensional (3D) image of the attenuation coefficients and the electron density distribution. Because X-ray PCCT is not yet clinically available, research is currently conducted on tissue specimens [17, 18]. A remarkable improvement of soft-tissue contrast without the use of contrast media has been demonstrated for different soft tissues such as liver, muscle and fat [14, 17]. Recently, quantitative PCCT imaging has been introduced and phase Hounsfield units (HUp) similar to the HUs in conventional attenuation-based imaging have been defined [14, 17, 19]. X-ray PCCT of cartilage is particularly challenging due to severe artifacts from adjacent bone in osteochondral samples [20]. However, it may be of particular importance in order to detect and quantify early biochemical cartilage matrix degeneration.

Purpose of this experimental study was (i) to demonstrate the feasibility of morphological high-resolution 3D X-ray PCCT cartilage imaging, (ii) to assess the performance of quantitative X-ray PCCT in comparison to T2 and T2* mapping at 3T MRI and T2 mapping at 7T MRI regarding the differentiation between healthy and degraded articular cartilage and (iii) to evaluate whether the different imaging techniques reflect biomechanical properties of articular cartilage. We hypothesized that grating-based X-ray PCCT may allow for high-resolution morphological and quantitative imaging of osteochondral samples and that all applied imaging techniques may contribute to an optimized multiparametric approach for cartilage tissue characterization in the context of early osteoarthritis.

## Methods

### Osteochondral samples

The study was approved by our institutional review board (Ethikkommission der Fakultät für Medizin der Technischen Universität München, Ismaninger Strasse 22, 81675 Munich, Germany) and all experiments were performed in accordance with relevant guidelines and regulations. Osteochondral samples (n = 12) of 6 mm in diameter were harvested from n = 6 from freshly slaughtered animals (femoral condyles of bovine knees; n = 2 plugs from each knee). The femoral condyles of bovine knees were “waste” retrieved from the butchers, that otherwise would have been disposed in the trash. The plugs were frozen at -20°C in phosphate buffered saline (PBS, Biochrom AG). Before experiments were conducted, samples were thawed to room temperature for at least 2 hours in phosphate buffered saline (PBS, Biochrom AG). One additional sample with a fissural morphological defect was scanned with PCCT and with morphological 3T and 7T MR sequences.

### Enzymatic cartilage degradation

To induce cartilage degradation, one of two samples from each knee was submerged in 2.5% Trypsin (Sigma-Aldrich). For controls, the other sample remained untreated and was immersed in Dulbecco’s Modified Eagle’s medium (DMEM, Gibco, Invitrogen). All specimens were incubated at 25°C for 24 hours. Cartilage depletion was stopped by removing the specimens from the enzyme solution and by washing the specimens with PBS. For the following experiments, the osteochondral samples were embedded in PBS.

### Biomechanical analyses

**Fig 1:**
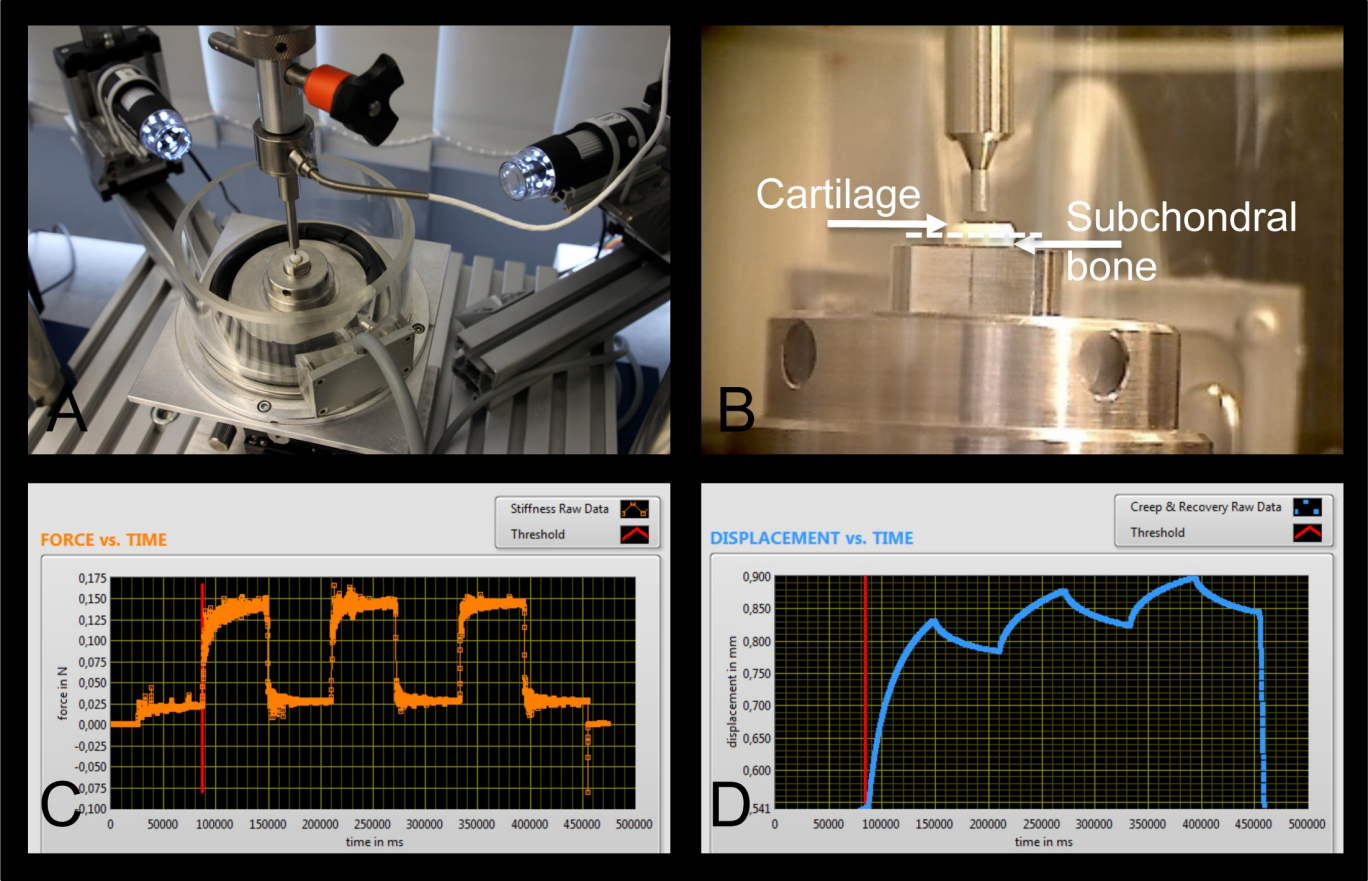
Principle of biomechanical creep indentation test. Cartilage stiffness was determined using a custom-made micro-indenter (A) and specimens were measured in a 4-DoF adjustable chamber filled with PBS (B), necessary to align the cartilage surface perpendicular to the indentation axis. The instantaneous stiffness response, indentation depth and recovery potential (backswelling) was measured during the loading ramp of the third creep cycle from the force (C) and the displacement (D) channels.

Cartilage stiffness measurements were conducted to verify that healthy and degenerated specimens provide a different mechanical behavior. This experimental verification was performed using a custom-made micro-indentation system (Fig 1A) [21]. The samples were clamped at their bone part by a rigid holder and the cartilage surface was individually aligned perpendicular to the indentation axis. For proper alignment two video cameras were placed at an angle of 90° to each other to directly inspect the orientation of the sample’s surface from two perpendicular directions (Fig 1B). Mechanical loading was applied using a creep/creep-recovery indentation protocol with a creep load of 0.11MPa [22] and a creep-recovery load of 0.005MPa. Three creep/creep-recovery cycles of 60s each were applied (Fig 1C) and position response was measured (Fig 1D). The load was distributed using a rigid, permeable indenter made of sintered titanium particles (outer diameter, 1.3mm; porosity, 50%; pore size, 100μm; SIKA-T, GKN Sinter Metals, Radevormwald/Germany). The first two loading cycles were used for preloading purposes. The instantaneous stiffness response, the creep indentation depth, and the recovery potential (the backswelling compared to the initial loading point) of the cartilage tissue were extracted from the third cycle. Stiffness was measured during the compression ramp, starting from 0.005MPa until reaching the creep load of 0.11MPa and a force controlled ramp speed of 0.1N/s. The linear part of the force-displacement curve was used to determine the instantaneous stiffness response of the cartilage by linear regression analysis. Additionally, creep indentation depth was defined by the strain value after 60s with respect to the original surface and thickness of the cartilage layer (normalized creep depth, given in % of the entire cartilage layer). Creep-recovery potential (backswelling of the tissue, %) values were evaluated using the same normalization procedure. Thickness of the samples was measured following the indentation experiments and after an additional recovery phase of 600s. Measurements were performed using the well-established needle indentation method [23, 24].

### 3T MR imaging

High resolution MR imaging at 3T was performed at a whole-body scanner (Ingenia, Philips Healthcare, Best, Netherlands) using an 8-channel wrist-coil. For morphological cartilage evaluation, the MR examination included a sagittal 2D intermediate (IM)–weighted (w) fat saturated (fs) turbo spin-echo (TSE) sequence (repetition time (TR), 10,000ms; echo time (TE), 50ms; matrix, 64x44; field of view (FOV), 32; slices, 32; slice thickness, 1.0 mm; gap 1.0mm; bandwidth, 94 Hz/pixel). For quantitative relaxation time measurements a sagittal 2D multi-slice multi-echo (MSME) spin-echo T2 mapping sequence (TR, 2200ms; TE, 5 echo times plus one simulated echo (16.6, 24.9, 33.1, 41.4, 49.7ms); matrix 140x139; FOV, 32; slices, 64; slice thickness 0.5mm; gap 0.5mm; bandwidth, 299 Hz/pixel) and a sagittal 3D MSME gradient-echo T2* mapping sequence (repetition time 122.8ms; 5 echo times plus one simulated echo (3.9, 7.3, 10.7, 14.0, 17.4ms); matrix 140x139; FOV, 32; slices, 64; slice thickness, 0.5mm; gap, 0.5mm; bandwidth, 438 Hz/pixel) were acquired.

### 7T MR imaging

High resolution MR imaging at 7T was performed at a horizontal bore 7T small animal scanner (Discovery MR901, GE Healthcare, Chalfont St. Giles, United Kingdom) using a 2-channel surface coil. For morphological cartilage evaluation, the MR examination included a 3D IM-w fs TSE sequence (TR, 3,000ms; TE, 20ms; matrix, 384x384; FOV, 60; slices, 72; slice thickness, 0.5mm; gap 0.5mm; bandwidth, 244 Hz/pixel). For quantitative relaxation time measurements, a sagittal 2D multi-slice multi-echo (MSME) spin-echo T2 mapping sequence was acquired (TR, 2000ms; TE, 8 echo times (10.6, 21.1, 31.7, 42.2, 52.8, 63.3, 73.9, 84.4ms); matrix 64x64; FOV, 30; slices, 66; slice thickness 0.5mm; gap 0.5mm; bandwidth, 299 Hz/pixel).

### X-ray phase-contrast imaging setup

**Fig 2:**
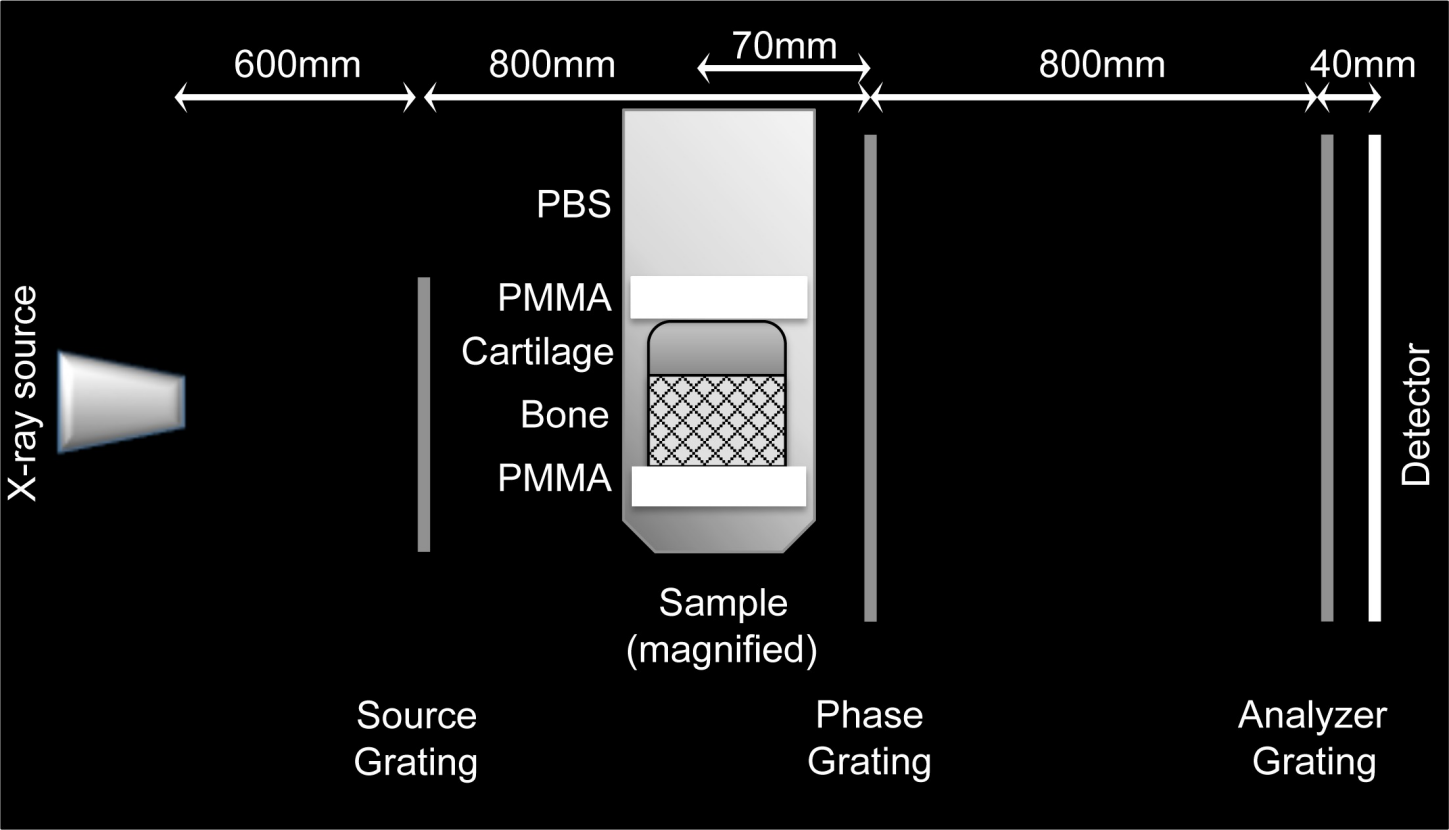
Sketch illustrating the setup of the X-ray phase contrast imaging system. A rotating anode X-ray tube and three different gratings were installed. Osteochondral samples were positioned perpendicular to the X-ray source.

The principle of grating-based X-ray PCCT and its data acquisition has been explained previously [14, 16, 17, 25, 26]. This imaging method does not only measure the attenuation of an X-ray beam, but it simultaneously measures its refraction (resulting in a phase shift) providing perfectly co-registered attenuation-contrast and phase-contrast images from the same acquisition. PCCT imaging was performed using the setup described in [27]. It consisted of a three grating Talbot–Lau interferometer in combination with an ENRAF Nonius FR591 rotating molybdenum anode X-ray tube and a single photon counting detector (Eiger, Dectris, Baden, Switzerland; silicon sensor thickness 450μm; 1030x1065 pixels, field of view 7.7x8.0cm^2^; effective pixel size 41x41μm^2^) [27, 28]. All three gratings had periods of 5.4μm (Institute of Microstructure Technology, Karlsruhe Institute of Technology, Karlsruhe, Germany) and the set consisted of two absorbing gold gratings and one phase grating. The duty cycles of the three gratings were approximately 0.6. One of the absorbing gold gratings was positioned directly behind the source, the other one in front of the detector (Fig 2). The third grating, a nickel phase grating, was placed at equal distance between the gold gratings (both inter-grating distances 857mm) and was designed for an effective energy of 27keV. The mean visibility of the interferometer reached approximately 29%. The rotation stage, holding and rotating the osteochondral sample, was mounted close to the phase grating (sample magnification, 1.7).

### Phase contrast imaging of collagen phantom and osteochondral samples

A calibration measurement was performed with PCCT on a collagen phantom consisting of 5 different collagen concentrations in gelatin. The concentrations varied from 0 to 20% wt (weight percent) in steps of 5% wt.

**Fig 3:**
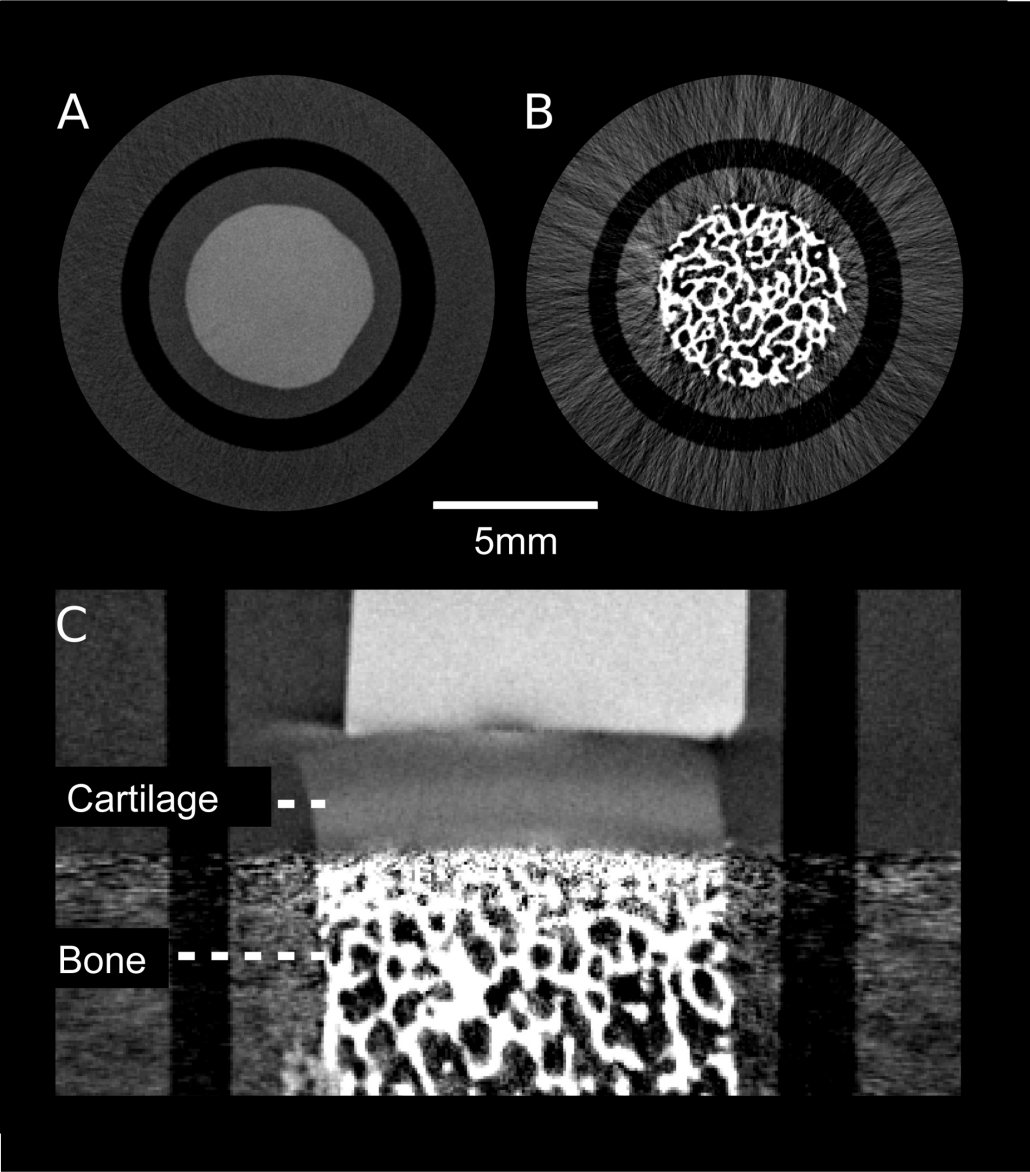
Phase contrast images. High-resolution three-dimensional phase contrast images were acquired axially, thus avoiding artifacts from the subchondral bone (B) to obscure the articular cartilage layer (A). Images were then reconstructed coronally for segmentation purposes (C). The white structure above the cartilage layer in C represents the polymethylmethacrylate (PMMA) rods for calibration of quantitative parameters.

Before PCCT measurements of osteochondral samples, the samples were again frozen at -20°C to account for long scan times (scan time 24 hours). Each sample was thawed to room temperature in PBS individually, before PCCT was conducted. The osteochondral samples were measured in an upright position (cartilage at the top and bone at the bottom) perpendicular to the X-ray beam (Fig 2) to reduce beam-hardening and X-ray scattering artifacts in the cartilage layer caused by the subchondral bone. The small tubes containing the osteochondral samples, polymethylmethacrylate (PMMA) rods for subsequent calibration of HUp and PBS [17], were placed in a water container [17, 29]. A full tomographic scan was acquired (tube voltage 40kVp; tube current 70mA) with 800 projections over 360°. For each projection 11 phase steps with 5s exposure time for each step were recorded. The high resolution used in this study also implied that the dose delivered to the samples is far beyond any clinical CT scan (in the range of several Gy). Thus, the dose level in this study is rather similar to the dose used for microCT. For the CT reconstruction an iterative algorithm (SIR) was used [30]. The reconstructed volumes (slice thickness, 41μm; number of slices, 460) were post-processed with a filter to reduce image noise [14]. Relative refractive index decrements from reconstructed 3D datasets were converted into HUp as described in Willner et al. [16, 17, 19, 31, 32]. The HUp of pure water was defined as 0HUp. Quantitative analysis in our investigations is based on the HUp values. The final data were digitally stored in DICOM format. For segmentation purposes, coronal reconstructions were obtained (Fig 3).

### Image analysis and cartilage segmentation

For visualization of cartilage morphology, MR examinations and PCCT examinations were transferred on picture archiving communication system (PACS) workstations (Easy vision, Philips, Best, Netherlands). T2 and T2* relaxation time maps were calculated pixelwise from MSME spin-echo images using a monoexponential non-negative least squares fit analysis with a custom-built software (IDL, Creaso, Gilching, Germany) [33]. For 7T MRI analyses, the first echo was excluded from the fitting process, in order to obtain more reliable values by eliminating the effects from stimulated echo signal on the calculated values [8, 34, 35]. For 3T MRI analyses, a first non-acquired echo was integrated in the MR sequence protocol to eliminate the effects from stimulated echo signal on the calculated values. Using the custom-built software, segmentation of artifact free cartilage areas was performed by placing regions-of-interest on every slice by one radiologist (T.B.). The superficial and the deep cartilage layer were segmented individually [36]; mean values were calculated for the entire cartilage layer. For PCCT, segmentation was performed similarly using OsiriX v.3.8.1 (32 bit). The collagen phantom data was evaluated using a custom-built software (Matlab, The Mathworks Inc, USA). The regions-of-interest were placed manually on every slice on homogeneous regions of each collagen concentration.

### Statistical analyses

Statistical processing was performed with SPSS version 20.0 (SPSS Institute, Chicago, IL, USA) (P.M.J., T.B.). All tests were performed based on a 0.05 level of significance. Means, standard deviations (SD), mean differences, standard errors of the mean (SEM) and 95% confidence intervals (CI) were calculated as indicated. Paired t-tests were used to compare quantitative cartilage values of degraded and healthy cartilage. Quantitative results from different imaging techniques were correlated with each other and with biomechanical parameters by using the Pearson correlation coefficient (R). The influence of different imaging and biomechanical parameters in one model was assessed using multivariate linear and logistic regression models. In addition, receiver operating characteristic (ROC) analyses were conducted. To compare the diagnostic accuracy between the different techniques in predicting cartilage degradation as a dichotomous outcome, the area under the receiver operating characteristic curve was used (calculation of AUC-values). An optimal cutoff value for each technique in the prediction of cartilage integrity as a dichotomous outcome was determined using the Youden-Index. Correspondingly, diagnostic performance with respect to detection of cartilage degradation including sensitivity and specificity was determined for all techniques.

## Results

### Characteristics of osteochondral samples

**Fig 4:**
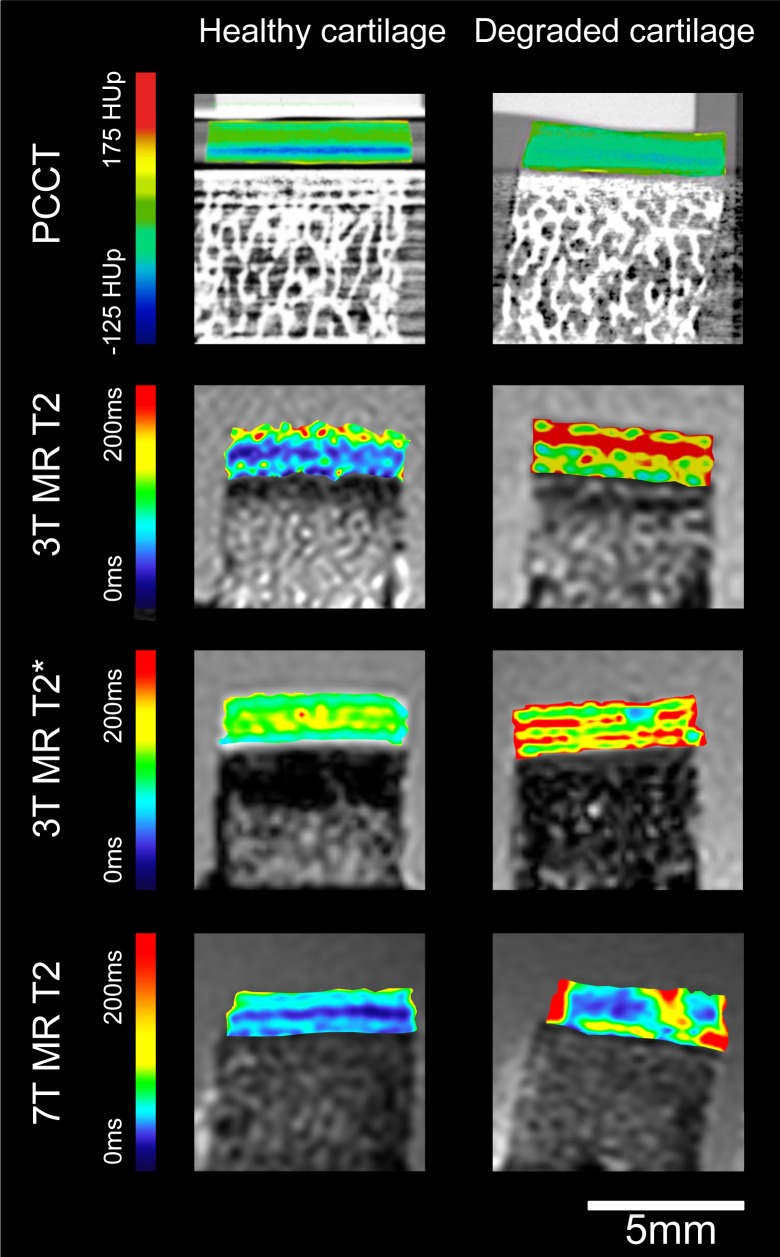
Color coded images for all imaging techniques of one healthy and one degraded osteochondral sample. For T2 and T2* images, color coded maps were overlaid with the first-echo images of the multi-echo sequence. Blue color indicates low, red color high cartilage quantitative values. Quantitative MR parameters showed statistically significant differences between the two groups. PCCT, Grating-based X-ray phase contrast computed tomography.

Osteochondral plugs had a mean cartilage thickness of 1.7±0.2mm and 1.6±0.1mm for healthy and degraded samples, respectively. A summary of quantitative results is presented in Table 1. Fig 4 shows exemplary color-coded images of healthy and degraded cartilage for each imaging technique.

**Table 1:** Averages ±SEM for different quantitative cartilage parameters in both groups.

Parameter	Healthy Cartilage	Degraded Cartilage	Mean Difference (95% CI)	P-value
*3T MRI T2 (ms)*	51.5 ±5.7	82.3 ±3.6	30.8 (16.2, 45.5)	0.003
3T MRI T2* (ms)	34.7 ±1.7	59.5 ±1.3	24.8 (21.2, 28.3)	<0.001
*7T MRI T2 (ms)*	42.8 ±1.6	80.9 ±6.0	38.0 (20.0, 56.1)	0.003
*PCCT (HUp)*	43.2 ±4.5	45.7 ±2.8	-2.4 (-9.9, 5.0)	0.439
*Stiffness (N/mm)*	18.8 ±2.5	9.8 ±0.8	-9.0 (-16.9, -1.1)	0.033
*Creep indentation (%)*	6.9 ±1.8	25.9 ±2.2	19.1 (14.2, 23.9)	<0.001
*Creep-backswelling (%)*	94.3 ±1.6	77.1 ±2.1	17.2 (12.7, 21.7)	<0.001

Healthy cartilage, untreated native cartilage; Degraded cartilage, cartilage with enzymatic degradation using trypsin; 95% CI, lower, upper 95% confidence interval; PCCT, Grating-based X-ray phase contrast computed tomography.

### Morphological evaluation

**Fig 5:**
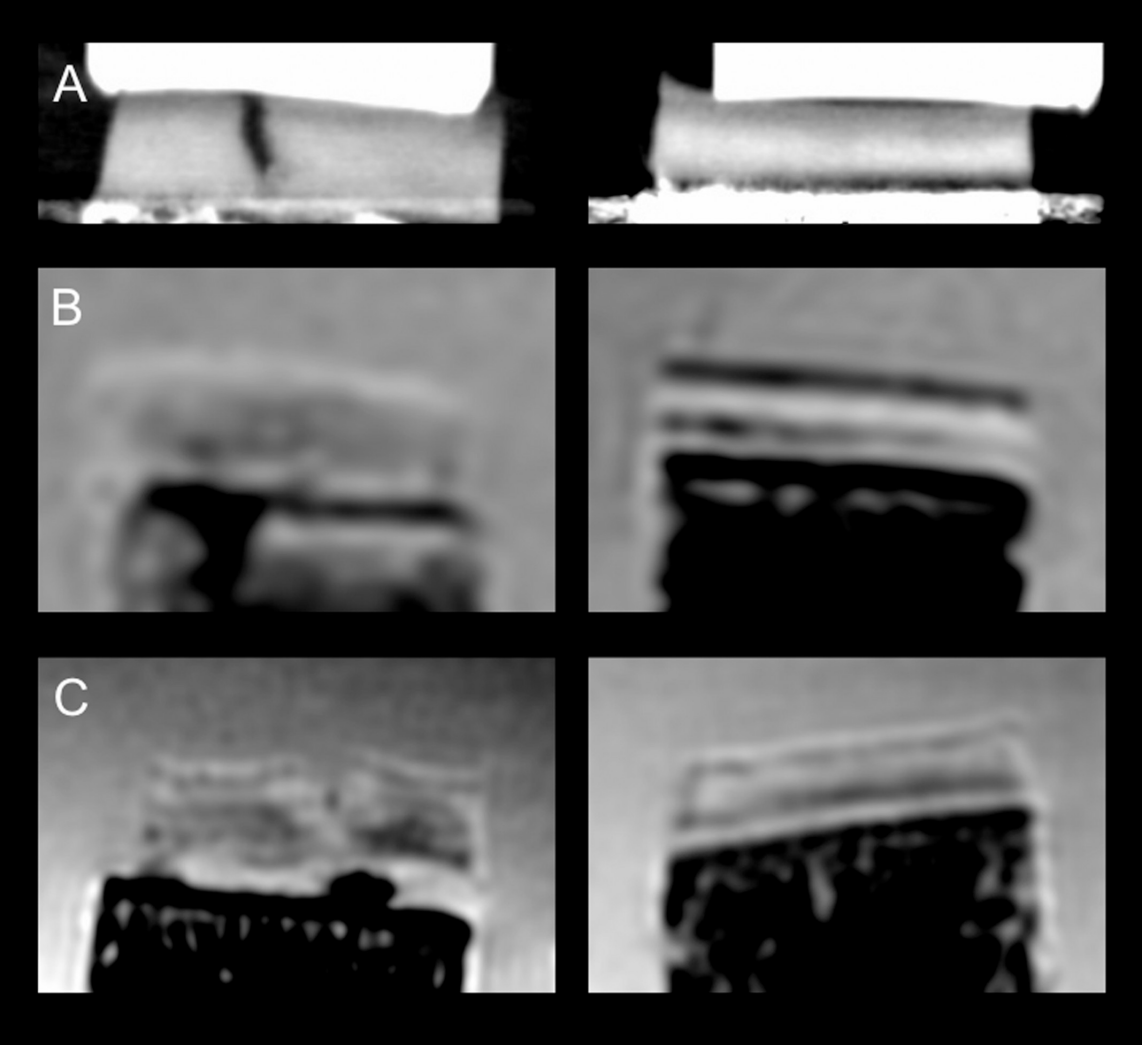
Morphological images of one osteochondral sample with a morphological cartilage defect (left column) and one intact osteochondral sample (right column) for all different imaging modalities. A: PCCT. B: 3T MRI, IM-w fs sequence. C: 7T MRI, IM-w fs sequence. The defect is clearly depicted on the PCCT image, while it may only be suggested on MR images.

In order to underline the high resolution of PCCT that allows improved morphological cartilage evaluation, one osteochondral sample with a morphological defect was scanned with PCCT and with morphological 3T and 7T MRI sequences. The morphological whole thickness fissural cartilage lesion was clearly depicted on the PCCT image, while it remained obscured on the 3T image and could be suggested on the 7T image (Fig 5).

### Biomechanical properties

In biomechanical indentation measurements, degraded cartilage samples showed a significantly reduced instantaneous stiffness response as compared to healthy cartilage (mean±SEM, 9.8±0.8N/mm versus 18.8±2.5N/mm; P = 0.033). The creep indentation depth was increased significantly in degraded samples (degraded cartilage, 25.9±2.2%; healthy cartilage, 6.9±1.8%; P<0.001). The recovery potential was also decreased significantly in degraded samples during the backswelling phase (degraded cartilage, 77.1±2.1%; healthy cartilage 94.3±1.6%; P<0.001).

### Cartilage MR imaging

At 3T, both T2 relaxation time measurements and T2* relaxation time measurements revealed significantly increased relaxation times for degraded cartilage samples. T2 values increased from 51.5±5.8ms to 82.4±3.4ms (means±SEM; P = 0.001). T2* values increased from 34.7±2.6ms to 63.0±1.4 ms (P<0.001). At 7T, T2 values increased from 43.4±3.6ms for healthy cartilage to 67.3±1.6ms for degraded cartilage (P = 0.001). The difference between superficial and deep cartilage layers was significant for 3T MRI T2 relaxation times of healthy and degraded cartilage (healthy: 68.0±7.0ms versus 35.0±.5.5ms, P = 0.001; degraded: 109.2±3.2ms versus 55.7±4.3ms, P<0.001) and 7T MRI T2 relaxation times of degraded cartilage (82.7±3.2ms versus 52.0±3.4ms, P = 0.003).

### X-ray PCCT collagen phantom and cartilage imaging

**Fig 6:**
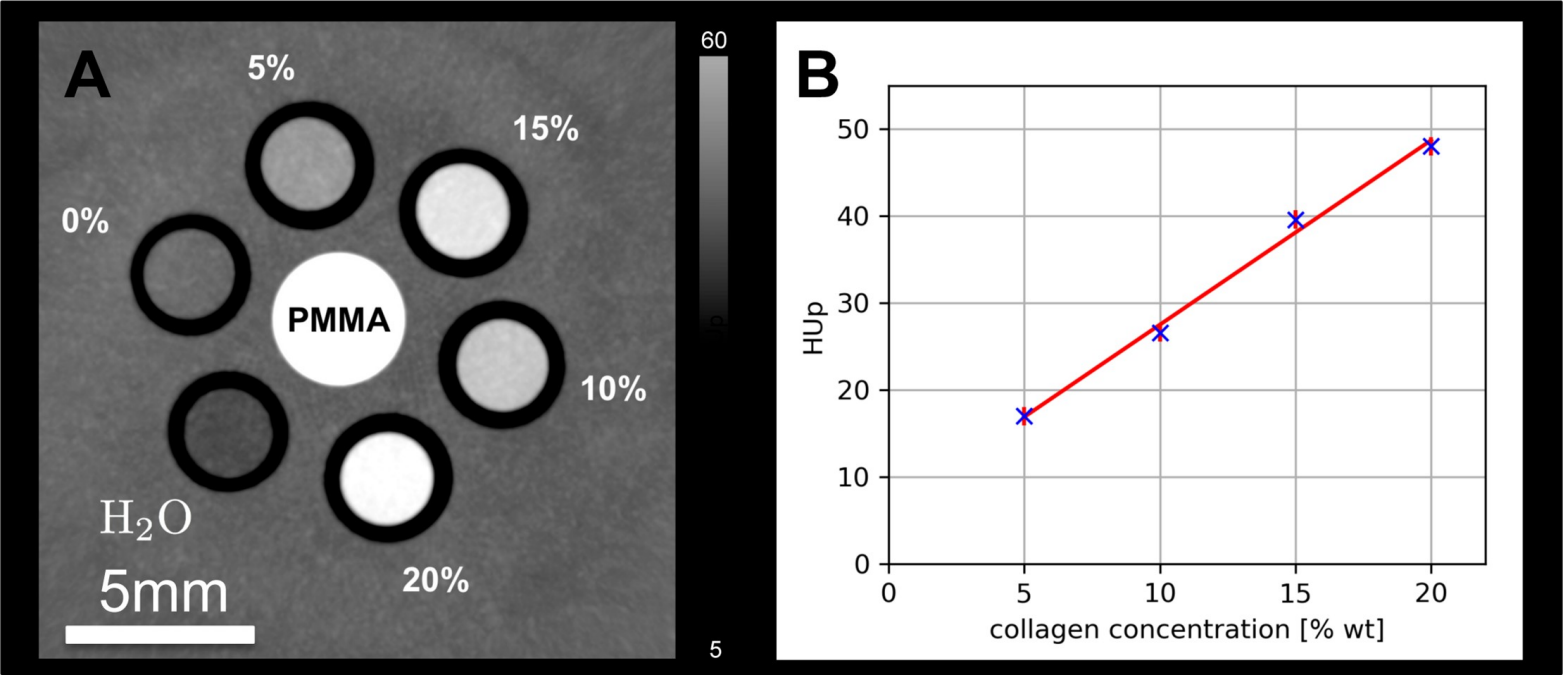
Collagen phantom. A: Phantom with different collagen contents (%) detected via Grating-based X-ray phase contrast computed tomography (PCCT). In the center a polymethylmethacrylate (PMMA) rod was positioned for calibration of quantitative parameters. B: Plot of HUps against the varying concentrations of collagen from the collagen phantom. PCCT was able to detect increasing concentrations of collagen.

The tomographic image of the collagen phantom and the results of the calibration measurement are shown in Fig 6. The grating-based X-ray PCCT was able to reliably detect increasing electron densities in correlation with increasing concentrations of collagen ranging from 5–20% wt.

Grating-based X-ray PCCT provided high-resolution images for improved morphological evaluation of native, non-decalcified osteochondral samples (Fig 3). Due to a dominating scattering signal caused by the porous bone structure and the high refractive index of calcium that prohibits the extraction of the phase, minor artifacts were depicted at the bone-cartilage-interface in the phase signal. Quantitative analyses provided phase Hounsfield unit (HUp) values. In all samples the deep cartilage layer showed significantly higher HUp values as compared to the superficial cartilage layer (35.9±1.8HUp versus 52.9±4.1HUp, P = 0.001). No significant differences between healthy (43.2±4.5HUp) and degraded (45.7±2.8HUp; P = 0.439) cartilage were found.

### Multivariate regression models

When including all imaging parameters and biomechanical parameters in one logistic regression model with the dichotomous outcome parameter healthy versus degraded cartilage, the influence remained significant for all parameters except for PCCT (P = 0.620): stiffness (P = 0.011), creep-indentation (P = 0.002), creep-backswelling (P = 0.002), 3T MRI T2 and T2* values (P = 0.004 and R = 0.001), 7T MRI T2 values (P = 0.002). In a multivariate linear regression model with stepwise backward regression, the outcome parameter creep indentation, 3T MRI T2 values and 7T MRI T2 values showed a significant influence, while the other parameters did not show an additional influence.

### Correlation analyses

Including all analyzed ostechondral samples in the correlation analyses, significant correlations were found between different quantitative MR parameters (Table 2). Cartilage creep-indentation and creep-backswelling correlated significantly with 3T T2, 3T T2* and 7T T2.

**Table 2:** Pearson correlations between different analyzed parameters pooling all analyzed samples.

Parameter	3T MRI T2*	*7T MRI T2*	*PCCT total*	*Stiffness*	*Creep- indentation*	*Creep-backswelling*
*3T MRI T2*	R = 0.91* (P<0.001)	R = 0.59* (P = 0.046)	R = -0.08 (P = 0.804)	R = -0.42 (P = 0.177)	R = 0.89* (P<0.001)	R = -0.89* (P<0.001)
3T MRI T2*		R = 0.78* (P = 0.003)	R = 0.14 (P = 0.679)	R = -0.64* (P = 0.024)	R = 0.94* (P<0.001)	R = -0.94* (P<0.001)
*7T MRI T2*			R = 0.44 (P = 0.152)	R = -0.88* (P<0.001)	R = 0.78* (P = 0.003)	R = -0.77* (P = 0.003)
*PCCT total*				R = -0.51* (P = 0.089)	R = 0.12 (P = 0.705)	R = 0.13* (P = 0.693)
*Stiffness*					R = -0.72 *(P = 0.009)	R = -0.72* (P = 0.009)
*Creep indentation*						R = 1.0* (P<0.001)

PCCT, Grating-based X-ray phase contrast computed tomography; Stiffness; Creep-indentation, indentation depth after 3^rd^ compression cycle (111kPa creep load); creep-backswelling, recovery capacity after 3^rd^ recovery cycle (5kPa test load).

*, P<0.05

### ROC analyses

Accuracy in predicting cartilage trypsination was excellent for quantitative MR imaging (AUC, 1.0), biomechanical stiffness measurements (AUC, 0.92), creep-indentation and creep-backswelling (AUC, 1.0) (Table 3). Accuracy in predicting cartilage trypsination was poor for quantitative PCCT HUp cartilage values (AUC, 0.56). Considering sensitivity and specificity as equally important, optimal cutoff values for the different parameters in the prediction of cartilage integrity were calculated and are given in Table 3. Using these cutoff values, high sensitivities and specificities of 100% were found for 3T T2, 3T T2* and 7T T2 values, creep-indentation and creep-backswelling.

**Table 3:** Area under the curve (AUC) determined with ROC analyses with respect to prediction of cartilage degradation and efficacy of different techniques in the detection of cartilage degradation.

**Statistic**	**3T MRI T2**	3T MRI T2*	**7T MRI T2**	**PCCT**
*AUC*	1	1	1	0.56
*95% CI*	1.00, 1.00	1.00, 1.00	1.00, 1.00	0.20, 0.91
*P-value*	<0.001	<0.001	<0.001	0.749
*Cut off value*	68.5 ms	51.9 ms	61.5 ms	46.8 HUp
*Sensitivity*	100%	100.00%	100.00%	67%
*Specificity*	100%	100%	100.00%	67%
**Statistic**	**Stiffness**	**Creep-indentation**	**Creep-backswelling**	
*AUC*	0.92	1	1	
*95% CI*	0.74, 1.00	1.00, 1.00	1.00, 1.00	
*P-value*	0.016	<0.001	<0.001	
*Cut off value*	11.6 ms	17.30%	84.90%	
*Sensitivity*	100%	100%	100%	
*Specificity*	75%	100%	100%	

95% CI, lower, upper 95% confidence interval; PCCT, Grating-based X-ray phase contrast computed tomography; pHU, phase contrast Hounsfield Units.

## Discussion

Non-contrast-enhanced grating-based X-ray phase-contrast computed tomography (PCCT) generates high-resolution morphological 3D images of osteochondral samples with a high soft-tissue contrast. Quantitative HUp values provide information on the collagen content of cartilage tissue and may therefore be of complementary use in addition to MRI and biomechanical features. MRI measurements at 3T and at 7T correlated significantly with biomechanical analyses and indicated cartilage softening. The diagnostic accuracy in the prediction of cartilage degradation was excellent for T2 and T2* relaxation time measurements, for biomechanical cartilage creep-indentation and creep-backswelling. The quantitative PCCT values were not significantly different between native and trypsinated cartilage. However, PCCT was able to reveal significant differences between the collagen content of the superficial cartilage layer and the collagen content of the deep cartilage layer. This lets assume, that while T2 relaxation time measurements do not only quantify collagen network integrity but also intracartilaginous water contents (which was increased by Trypsin treatment), PCCT may be more specific with respect to detection of extracellular matrix changes, particularly collagen contents, while ignoring water contents. This multimodal concept may provide an ideal non-invasive approach for optimized high-resolution morphological and quantitative cartilage tissue imaging and improved cartilage biomatrix characterization.

Hyaline cartilage primarily consists of extracellular matrix, including collagen (15–20%), proteoglycans (3–10%), and water (80%), and only to about 1% of chondrocytes [7]. The combination of these components provides the important viscoelastic properties, that are essential for proper cartilage function [6]. Early cartilage degeneration is characterized by collagen disruption and loss of proteoglycans, which induces an increase in cartilage water content and mobility [7]. Cartilage degeneration leads to early osteoarthritis [1, 2, 4, 37]. New molecular MR imaging biomarkers are able to detect and quantify early biochemical changes of the cartilage matrix before morphological defects occur [3, 7, 8, 38]. dGEMRIC and T1rho relaxation time measurements were described to correlate with proteoglycan contents. However, clinical applications are limited, since dGEMRIC requires contrast administration and for T1rho measurements specific absorption rate (SAR) issues were described [5, 8]. T2 relaxation time measurements are clinically feasible and primarily correlate with collagen disruption and increasing water contents [39]. In our study, we used the trypsin for induction of cartilage degradation, as performed by previous groups [40]. Trypsin is known to primarily degrade proteoglycans and to not affect collagen distribution. Despite, at 3T and at 7T, T2 relaxation time measurements showed an excellent accuracy in predicting trypsin induced cartilage degradation in our study. T2 values were significantly increased in degraded cartilage samples possibly due to increases in water contents, as described previously by Nissi et al., who found a trend to a correlation of T2 with cartilage water contents [41]. The authors also stated that most MRI parameters were sensitive to both glycosaminoglycan content and collagen network integrity. T2* relaxation time measurements were described to be more sensitive for cartilage degradation in the deep layer [5]. In our study, biomechanical analyses were in line with MRI measurements at 3T und 7T and confirmed the softening of cartilage tissue after trypsin treatment. PCCT revealed to be insensitive to increasing water content in cartilage, which is due to the comparably low electron density of water compared to collagen. On the contrary, experimental collagen concentration measurements and differences between the superficial and the deep cartilage layer indicate that PCCT was able to detect collagen contents and collagen matrix density. Therefore quantitative HUp values may be able to reliably monitor changes in collagen contents in cartilage tissue without a confounding water sensitivity. PCCT may provide more detailed information on the underlying biochemical causes of cartilage degeneration.

Quantitative MR biomarkers, including T2 relaxation time measurements, were described to non-invasively predict functional, biomechanical properties of articular cartilage [12, 42, 43]. Most previous studies that correlate biomechanical properties with quantitative cartilage MR imaging were performed at 9.4T or at 1.5T. In contrast to 3T and 7T scanners [44], there are only few 9.4T scanners, which strongly limits clinical applications. In the present study, T2 values were lower at 7T than at 3T, which is in line with previous findings [44]. In biomechanical analyses, T2 mapping correlated significantly with the indentation creep and creep-recovery. Assessing naturally degraded cartilage at 3T, Juras et al. did not find significant correlations of T2 relaxation times with biomechanical properties [42]. For 9.4T imaging, Rautiainen et al. reported that T2 values were able to differentiate between early and advanced osteoarthritis and that T2 values correlated with histological results and mechanical properties [11]. While in most other studies no enzymatically degraded cartilage was used, in a recent study Nissi et al. found statistical trends for the correlation of 9.4T T2 values with Young’s modulus and with cartilage water contents in enzymatically degraded cartilage samples [41]. No study determined accuracy of the techniques in order to predict cartilage degradation using ROC analyses.

In addition to established MR imaging, we examined the new cross-sectional phase contrast imaging technique for evaluation of osteochondral samples. Besides an improved soft tissue contrast a complimentary quantitative value (the distribution of electron density correlating with the collagen content) is another advantage of the phase contrast in comparison to conventional CT imaging [17]. In parallel to further improvements of the technique, establishing possible applications for different tissues is mandatory. Quantitative techniques for evaluation of tissue contrast and its decomposition in lipid, protein and water content have recently been introduced [17, 45]. High-resolution morphological assessment and quantitative evaluation of different soft tissues including muscle, fat, and skin were described. Also, trabecular bone has successfully been imaged using grating-based phase contrast imaging [46, 47]. However, cartilage imaging of osteochondral tissue has been impossible due to severe overlying artifacts from the bone component. Recent improvements of phase contrast imaging techniques including filtering and iterative reconstruction allowed visualization of the cartilage layer in osteochondral samples in this study [17, 45]. Using the grating-based x-ray phase contrast imaging technique, two-dimensional (2D) morphological imaging of articular cartilage has previously been performed. Marenzana et al. were able to image small cartilage lesions in rat cartilage [48]. Tanaka et al. developed an X-ray phase imaging system based on Talbot-Lau interferometry and studied its feasibility for clinical diagnoses of joint diseases [49]. The results already indicated sufficient sensitivity to cartilage, suggesting medical significance [49]. Horn et al. used this technique to image cadaveric knees and showed that chondrocalcinosis of the meniscus could more evidently be detected dark-field image in comparison to the conventional attenuation image [50]. Momose et al. succeeded to image cartilage in finger joints [51, 52]. In our study, the resolution of 41μm was superior to previously published images and to high-resolution MR images. For the first time, quantitative values for measurements of cartilage collagen contents were provided. The biomechanical parameters and T2 relaxation times confirmed the Trypsin induced cartilage degradation, most likely due to resulting higher water content, while PCCT showed a stable collagen content after degradation. Using PCCT, a significant gradient in the collagen content decreasing form deep to superficial layer was demonstrated. MR imaging and PCCT examine different physical effects and therefore provide different, complementary information on cartilage tissue composition. Additionally, PCCT allows for high-resolution morphological evaluation of cartilage integrity. Further studies that combine MRI and PCCT are mandatory to fully understand the artificial degradation.

Since this was a preliminary feasibility study, there are several limitations. Currently, PCCT scanners are only experimental and therefore only small ex vivo specimens could be analyzed. In this preliminary study, the sample number was small due to high resolution imaging that requires long PCCT scan times. One reason for the scan times is the long distance between the x-ray source and the source grating, yielding decreased flux at the detector. Further, healthy and degraded osteochondral sample were not identical. However, recruiting one healthy and one degraded sample from each bovine knee accounts for interindividual differences in cartilage ultrastructure. This study basically included intact cartilage layers. Only one sample with a fissural cartilage defect was scanned exemplarily. In future studies the qualitative superiority of high resolution PCCT with respect to detection of morphological cartilage defects needs to be confirmed. Last, further investigations including collagenase treatments, histological correlations, and other quantitative MR imaging sequences are needed.

## Conclusion

In summary, biomechanical analyses confirmed the softening (e.g. degradation) of the cartilage samples after Trypsin treatment. 3T and 7T T2 values correlated significantly with biomechanical properties. Feasibility of high-resolution grating-based x-ray PCCT imaging of native non-decalcified osteochondral samples was demonstrated successfully. In addition to improved morphological evaluation, quantitative HUp values may be able to monitor the collagen content in healthy and degraded cartilage. Quantitative X-ray phase-contrast imaging may therefore become a valuable, complementary tool with respect to cartilage characterization. This multimodal concept may provide an ideal non-invasive multimodal approach for optimized high-resolution cartilage imaging, and improved characterization of osteochondral tissue in the context of early osteoarthritis.
